# Efficacy and Safety of *Salvia miltiorrhiza* Extract (SAGX) Compared with Saw Palmetto in Men with Lower Urinary Tract Symptoms: A 12-Week, Randomized, Double-Blind, Parallel-Group Pilot Study

**DOI:** 10.3390/nu18111752

**Published:** 2026-05-29

**Authors:** Eun Young Kim, Eun Joo Lee, Joon Young Chang, Sang Jun Im, Young Ho Park, Ha Young Kim

**Affiliations:** 1 Gangnam Guard Urology Clinic, Seoul 07008, Republic of Korea; key9326@naver.com; 2 College of Pharmacy, CHA University, Pocheon 11160, Republic of Korea; ejlee1109@chauniv.ac.kr; 3 Curome Biosciences Co., Ltd., Suwon 16229, Republic of Korea; mtupr_77@curome.co.kr (J.Y.C.); cocohut@naver.com (S.J.I.); yhpark@curome.co.kr (Y.H.P.)

**Keywords:** lower urinary tract symptoms, *Salvia miltiorrhiza*, international prostate symptoms score, international index of erectile function, SAGX

## Abstract

**Background:** Lower urinary tract symptoms (LUTS) are prevalent among aging men and negatively affect quality of life. *Salvia miltiorrhiza* extract (SAGX), which exhibits anti-inflammatory and antioxidant properties, has been developed as a functional ingredient to improve LUTS. However, comparative clinical data against established standards, such as saw palmetto, are currently lacking. **Objectives**: To compare the efficacy and safety of SAGX with saw palmetto extract in men with LUTS. **Methods**: In this randomized, parallel-group pilot study, 30 men with LUTS were assigned to receive either 400 mg of SAGX or 320 mg of saw palmetto extract once daily for 12 weeks. The primary outcome was the change in the International Prostate Symptom Score (IPSS) and the LUTS-related quality-of-life (QoL) score. Secondary outcomes included changes in the erectile function assessed using the International Index of Erectile Function (IIEF). Safety was evaluated by monitoring adverse events. **Results**: SAGX supplementation resulted in a significantly greater reduction in total IPSS compared with the saw palmetto group (*p* = 0.031), with notable improvements in storage symptoms (*p* = 0.003) and QoL (*p* = 0.035). Erectile function also improved significantly in the SAGX group (*p* = 0.005). All adverse events were mild and transient, with treatment-related events occurring less frequently in the SAGX group. **Conclusions**: Although this study was a pilot study with a limited cohort, these findings provide preliminary evidence for the use of SAGX as a functional ingredient for supporting urinary health in men with LUTS.

## 1. Introduction

Lower urinary tract symptoms (LUTS) represent one of the most common and troublesome health concerns among aging men and affect over 1 billion elderly men globally [1,2]. Characterized by urinary frequency, nocturia, urgency, weak stream, and incomplete bladder emptying, these symptoms can disrupt daily activities, diminish confidence, and significantly reduce quality of life [3]. Although LUTS may arise from various lower urinary tract and prostate-related conditions, benign prostatic hyperplasia (BPH) is reported to be one of the most common causes in elderly men [4,5].

Conventional pharmacotherapies—such as α-adrenergic antagonists (e.g., tamsulosin, alfuzosin) and 5-α-reductase inhibitors (e.g., finasteride, dutasteride)—are effective in improving LUTS, but can be associated with undesirable side effects. α-Blockers may cause ejaculatory disorders, dizziness, and orthostatic hypotension, while 5-ARIs have been linked to reduced libido, erectile dysfunction, and decreased ejaculate volume, with some reports of persistent sexual dysfunction even after discontinuation [3]. These drawbacks frequently discourage long-term adherence and have fueled interest in safer, plant-based interventions that maintain symptom relief without diminishing quality of life.

Saw palmetto (*Serenoa repens*) extract has been widely used for decades as a representative supportive plant-based health remedy for LUTS. However, the findings from larger and more methodologically rigorous randomized controlled trials have been mixed, leading to ongoing discussions regarding the extent of its clinical benefits. In the STEP trial, administration of saw palmetto extract at 160 mg twice daily for 12 months did not demonstrate clear differences from the placebo in the American Urological Association International Prostate Symptom Score (AUA/IPSS), peak urinary flow, or prostate volume. Similarly, the CAMUS study reported that even with dose escalation up to 960 mg/day over 72 weeks, improvement in LUTS was not consistently observed across clinical endpoints [6,7,8,9,10]. Contemporary Cochrane reviews and recent meta-analyses including over 4000 men generally suggest that saw palmetto monotherapy may provide limited symptomatic benefit for LUTS associated with benign prostatic enlargement, although some uncertainty remains regarding the potential role of specific hexanic extracts or combination formulations [11]. These findings highlight an important limitation of current supportive plant-based strategies for LUTS, many of which primarily target androgen signaling pathways. Increasing evidence suggests that oxidative stress, chronic inflammation, and pelvic microvascular dysfunction also contribute substantially to LUTS pathophysiology [12,13,14,15]. Consequently, plant-derived compounds that target these mechanisms may provide a complementary or alternative nutritional approach.

*Salvia miltiorrhiza*—also known as Danshen or red sage—is one of the cornerstone herbs in traditional East Asian medicine and has gained increasing scientific attention for its anti-inflammatory, antioxidant, vasodilatory, and cytoprotective properties [16,17,18]. Its application spans centuries and has traditionally been used for cerebrovascular and cardiovascular disorders owing to its antioxidative and anticoagulative effects [19,20].

The therapeutic potential of *S. miltiorrhiza* is primarily attributed to its lipophilic constituents, specifically a group of compounds known as tanshinones. These compounds have been shown to modulate mitochondrial bioenergetics, reduce oxidative stress, and attenuate inflammatory signaling cascades implicated in prostatic and urinary tract dysfunction [16,18]. SAGX is a proprietary extract uniquely standardized to concentrate these lipophilic bioactive components, with a mandatory threshold of at least 10% cryptotanshinone serving as a quality control marker for the total tanshinone complex. Preclinical studies have demonstrated that this specific tanshinone-rich profile exerts potent antioxidant activity, enhances nitric oxide-mediated smooth muscle relaxation, and activates the Nrf2-HO-1 cytoprotective pathway. Collectively, these effects have been shown to improve urinary flow and support pelvic organ function in animal models, providing the mechanistic rationale for the current clinical pilot study [17].

In support of these findings, a recent clinical study in Korean men with LUTS reported significant reductions in symptom severity and improvements in quality-of-life (QoL) scores following SAGX supplementation [21]. Moreover, SAGX has been approved by the Korean Ministry of Food and Drug Safety (MFDS) as a functional ingredient that “may help to maintain a healthy prostate.” These findings suggest that SAGX holds similar potency to saw palmetto as a plant-based nutritional option for the management of LUTS. While these data are promising, head-to-head comparative trials between SAGX and the supportive nutritional standard, saw palmetto, are currently lacking. Furthermore, the relative efficacy and clinical utility of SAGX within the broader spectrum of nutritional options for men’s urological health remain to be clarified.

The present study aimed to evaluate and compare the efficacy and safety of SAGX to saw palmetto extract in a population of elderly male patients with LUTS. The study was designed as a randomized, double-blind, parallel-group pilot study over 12 weeks. We assessed changes in LUTS using the IPSS as the primary outcome. To explore potential effects on sexual well-being, the International Index of Erectile Function (IIEF) was included as a secondary outcome measure.

## 2. Materials and Methods

### 2.1. Study Design

This study was designed as a 12-week, single-site, randomized, double-blind, parallel-group pilot study. The study was conducted at Gangnam Guard Urology Clinic, Seoul, Republic of Korea, between May and October 2025. The protocol was approved by the Public Institutional Review Board of the Korea National Institute for Bioethics Policy under the Ministry of Health and Welfare, and registered in the Clinical Research Information Service (CRIS) of South Korea.

Following screening, eligible subjects who met all inclusion and exclusion criteria were randomized in a 1:1 ratio to receive either SAGX or saw palmetto extract, using a block randomization method prior to the initiation of the study. The study was conducted under double-blind conditions, with all study personnel, including the principal investigator (PI), subjects, and study staff, remaining blinded to treatment allocation.

Clinical oversight and the administration of patient-reported outcome measures were performed by a board-certified urologist with over 25 years of clinical practice. The PI holds full memberships in the Korean Urological Association (KUA), the Korean Prostate Society (KPS), and the International Society for Sexual Medicine (ISSM), among others, ensuring that all diagnostic evaluations and the validation of symptom scores followed established urological guidelines and Good Clinical Practice (GCP).

### 2.2. Investigational Products

SAGX is a dried ethanol extract of the root of *S. miltiorrhiza* and is standardized to contain a minimum of 10% cryptotanshinone as its marker compound. The intake dose was determined based on a previous dose-finding study in male patients with LUTS, where both the 400 mg and 800 mg daily doses demonstrated comparable efficacy over 12 weeks [21]. Consequently, the 400 mg dose (2 capsules) was selected for the present study to optimize patient compliance and safety. Saw palmetto extract was administered at a standard daily dose of 320 mg (2 capsules) for 12 weeks, in accordance with established clinical protocols [7].

Both the SAGX and saw palmetto extract were encapsulated and provided by Novarex Co., Ltd. (Cheongju, Republic of Korea), a facility certified for Good Manufacturing Practices (GMP) by the Ministry of Food and Drug Safety (MFDS). To ensure the integrity of the double-blind design, both substances were provided in identical unlabeled soft capsules that were indistinguishable in size, shape, and appearance. The concentration of the active principles for both extracts was verified for each batch prior to encapsulation to ensure batch-to-batch consistency. The tanshinone content of SAGX was determined via high-performance liquid chromatography (HPLC), while the fatty acid profile of the saw palmetto extract, including a minimum of 220 mg/g of lauric acid, was verified via gas chromatography (GC) in accordance with the MFDS standardized test methods.

### 2.3. Study Population

To date, there has been no study directly comparing SAGX and saw palmetto in subjects with lower urinary tract symptoms. This study was designed as an exploratory pilot trial to compare SAGX and saw palmetto. Accordingly, a formal sample size calculation based on predefined hypotheses and statistical power was not performed, as the primary objective was to assess feasibility and obtain preliminary estimates of variability. Instead, a target sample size of 12 participants per group was selected, in line with commonly recommended sample sizes for pilot studies [22]. Accounting for an anticipated 20% dropout rate, the total enrollment goal was set at 15 participants per group (30 total).

#### 2.3.1. Inclusion Criteria

Eligible participants were men aged 40–80 years with a clinical diagnosis of mild to moderate LUTS and who provided written informed consent prior to enrollment. The diagnosis of mild to moderate LUTS was established by a board-certified urologist based on an IPSS of 8–19 and confirmed via standard clinical assessments, including prostate ultrasound and uroflowmetry.

#### 2.3.2. Exclusion Criteria

(1) Presence of severe cardiovascular, immunological, respiratory, hepatobiliary, renal/urinary, neurological, musculoskeletal, psychiatric, infectious, or malignant diseases, unless deemed eligible by the principal investigator; (2) prostate-specific antigen (PSA) ≥ 4.0 ng/mL, unless malignancy was ruled out within 3 months of Visit 1; (3) a diagnosis within 4 weeks prior to Visit 1 of urinary tract stones, urethral strictures, bladder neck contracture, lower urinary tract inflammation (bladder or urethra), urinary tract tuberculosis, prostatitis, urinary tract infection, acute urinary retention, or neurogenic bladder; (4) a history of prostate or bladder cancer; (5) a history of prostate-related surgery or other invasive procedures; (6) a history of surgery affecting lower urinary tract function (e.g., urethrotomy or bladder neck incision); (7) uncontrolled hypertension (systolic blood pressure > 160 mmHg or diastolic blood pressure ≥ 100 mmHg after 10 min of rest); (8) uncontrolled diabetes (fasting glucose ≥ 180 mg/dL or initiation of antidiabetic therapy within 3 months); (9) thyroid disease; (10) use of medications for benign prostatic hyperplasia (BPH) or related functional foods, unless on a stable dose for ≥4 weeks with no expected changes during the study, at the investigator’s discretion; (11) aspartate aminotransferase (AST) or alanine aminotransferase (ALT) > 3× the upper limit of normal; (12) coagulation disorders, anemia, abnormal activated partial thromboplastin time (aPTT), or use of anticoagulant medications; (13) participation in another interventional clinical trial within 3 months of Visit 1 or planned participation during the study; (14) known hypersensitivity to the study-related food ingredients; (15) any condition deemed by the investigator to render the participant unsuitable for the study; (16) study site personnel.

Following these criteria, lifestyle factors and concomitant medications were monitored throughout the study period. While alcohol consumption and tobacco use were not grounds for exclusion, they were meticulously documented in the case report forms (CRF). To reflect a realistic LUTS patient population, subjects with managed co-morbidities—such as stable hypertension or hyperlipidemia—were permitted to continue their established treatment regimens, provided that the dosage and medication remained unaltered for the 12-week duration. Baseline analysis of the randomized safety set (*N* = 30) confirmed that these lifestyle and clinical factors were statistically balanced between the SAGX and saw palmetto groups.

### 2.4. Outcome Measures

The primary outcome measure was the mean change in the International Prostate Symptom Score (IPSS) and the LUTS-related quality-of-life (QoL) score from the baseline to the 12-week endpoint. The IPSS is a validated, self-administered diagnostic tool used to grade the severity of LUTS [23]. Questionnaires were obtained from the participants at baseline and Week 12, and consisted of the following categories.

- Symptom Assessment: seven questions covering incomplete emptying, frequency, intermittency, urgency, weak stream, hesitancy, and nocturia.
- Quality-of-life (QoL) score: a single question assessing the patient’s perceived QoL regarding their urinary symptoms.
- Scoring: each symptom is scored from 0 (not at all) to 5 (almost always), with total scores ranging from 0 to 35. Scores are typically categorized as mild (≤7), moderate (8–19), or severe (20–35).
- Voiding and storage subscores: the IPSS can be further subdivided into voiding and storage subcategories. The voiding subscore (0–20) includes incomplete emptying, intermittency, weak stream, and straining, while the storage subscore (0–15) includes frequency, urgency, and nocturia.

The secondary outcome measure was determined as the change in total International Index of Erectile Function (IIEF) score after 12 weeks of treatment. The IIEF evaluates five distinct domains of male sexual function: erectile function, orgasmic function, sexual desire, intercourse satisfaction, and overall satisfaction [24]. Questionnaires were completed by participants at the baseline and 12 weeks. The questionnaires are included in Supplementary Materials S1 (IPSS) and S2 (IIEF).

### 2.5. Safety and Adverse Events

Safety was rigorously monitored throughout the 12-week study period. Safety monitoring was conducted through a hybrid schedule of 3 to 5 touchpoints per subject, including three scheduled in-person visits and supplemental monitoring via telephone or SMS at 3- to 4-week intervals. To ensure comprehensive data collection, investigators utilized an active surveillance approach featuring open-ended questioning and feedback summarization in a comfortable clinical environment. This method was designed to capture even minor symptomatic changes, which were then documented in the case report forms (CRFs) including onset, resolution, and causality assessment.

An adverse event (AE) was defined as any untoward medical occurrence in a participant, regardless of whether it had a causal relationship with study treatment.

- Monitoring protocol: participants were monitored via the hybrid schedule described above; all reported physiological or psychological changes were systematically evaluated by the investigator during each touchpoint.
- AE classification and causality assessment: AEs were graded by severity (mild, moderate, or severe) and evaluated for their “relatedness” to the study intervention (definitely not related, probably not related, possibly related, probably related, or definitely related).
- AE management: in the event of a serious adverse event (SAE), the principal investigator (PI) retained the authority to unblind the participant or discontinue their involvement to ensure patient safety, following standard Good Clinical Practice (GCP) guidelines.

### 2.6. Data Analysis

Efficacy analyses in this exploratory pilot study were conducted using the per-protocol set (PPS), defined as participants who completed the full 12-week intervention with a treatment compliance of ≥80% and without major protocol deviations. The primary efficacy endpoint was the change in total International Prostate Symptom Score (IPSS) and QoL score from baseline to Week 12. In the case of missing values for efficacy endpoints, the last observation carried forward (LOCF) method was utilized. Missing data for non-efficacy variables were not imputed and were analyzed as observed.

Given the small sample size and non-normal distribution of the data, non-parametric methods were applied. Within-group changes in IPSS and International Index of Erectile Function (IIEF) scores were analyzed using the Wilcoxon signed-rank test, while between-group comparisons were performed using the Wilcoxon rank-sum test (Mann–Whitney U-test). Non-parametric tests were specifically chosen to ensure statistical validity in the presence of the high inter-individual variability commonly observed in subjective urological symptom reporting. Descriptive statistics were used to summarize the study data, with continuous variables presented as the mean ± standard deviation and categorical variables as frequencies and percentages. Statistical significance was assessed using two-sided tests with a significance level of 0.05. Given the exploratory nature of the study, no adjustment for multiple comparisons was performed.

Demographic and baseline clinical characteristics were summarized to describe the study population. Between-group comparisons for categorical variables were conducted using the chi-square test or Fisher’s exact test, as appropriate. If statistically significant differences in baseline characteristics between groups were identified, a general linear model (GLM) was applied with the relevant covariates to adjust for potential confounding effects. Extreme values for continuous variables were handled according to predefined criteria.

## 3. Results

### 3.1. Baseline Characteristics and Participant Distribution

A total of 33 individuals were screened for eligibility, of whom 3 were excluded for not meeting the inclusion criteria. The remaining 30 participants were randomized.

In the SAGX group, 2 of the 15 randomized participants discontinued the study, resulting in 13 participants completing the trial. In the saw palmetto group, 3 of the 15 randomized participants discontinued, with 12 participants completing the study.

**Figure 1:**
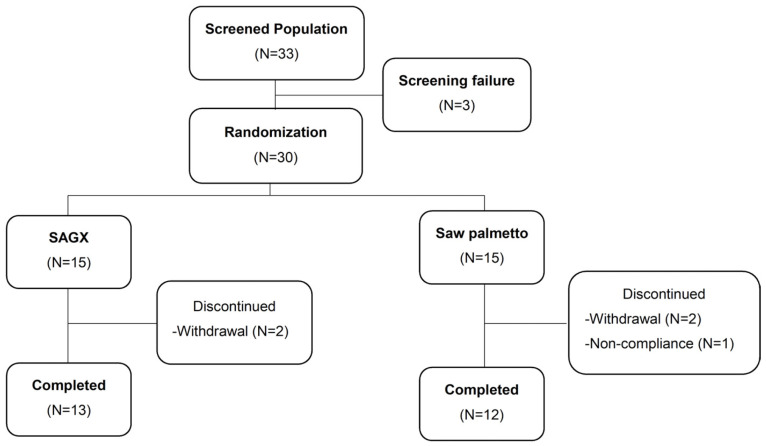
CONSORT diagram of patient disposition.

Participant flow is summarized in the CONSORT diagram (Figure 1).

The mean age of the participants was 67.0 ± 6.8 years in the SAGX group and 67.4 ± 6.9 years in the saw palmetto group, with no statistically significant difference between groups (*p* = 0.473, Wilcoxon rank-sum test). Height, weight, and blood pressure (both systolic and diastolic) also showed no statistically significant differences between the two groups (Table 1).

### 3.2. Primary Endpoints

#### 3.2.1. IPSS Total Score

**Figure 2:**
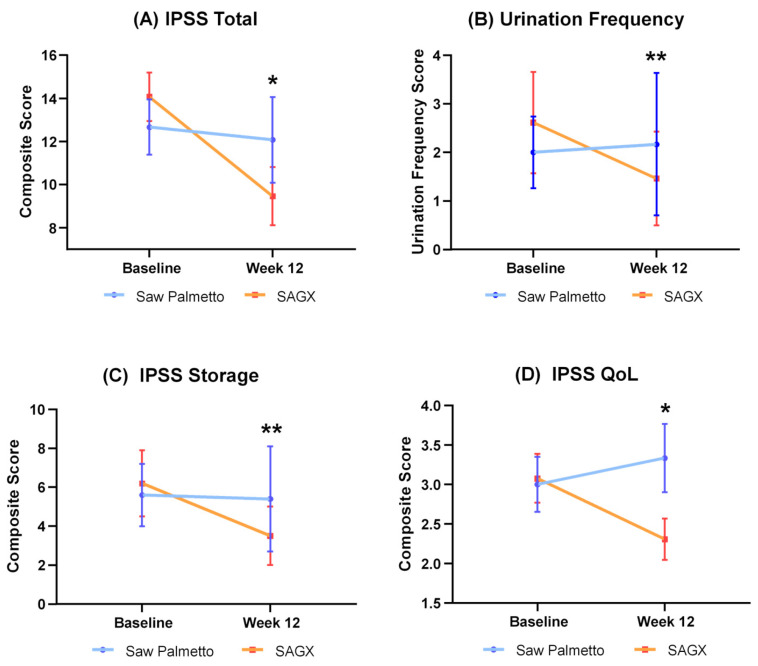
Changes in International Prostate Symptom Score (IPSS) at 12 weeks (PPS, mean ± SEM; vertical red and blue lines represent SEM). (**A**) IPSS total score from baseline to Week 12 (*p* = 0.031). (**B**) Urination frequency from baseline to week 12 (*p* = 0.006). (**C**) IPSS storage from baseline to Week 12 (*p* = 0.003). (**D**) IPSS quality-of-life (QoL) score from baseline to Week 12 (*p* = 0.035). * *p* < 0.05, ** *p* < 0.01, based on between-group change from the baseline (Δ12 wk), via the Wilcoxon rank-sum test. Lower scores indicate improvement.

At 12 weeks, the SAGX group demonstrated a significantly greater reduction in total IPSS score compared with the saw palmetto group (Figure 2A). The mean IPSS score decreased from 14.1 ± 4.1 at baseline to 9.5 ± 4.9 at Week 12 in the SAGX group, whereas the saw palmetto group showed a more modest reduction from 12.7 ± 4.4 to 12.1 ± 6.9.

The mean change in total IPSS (Δ12 wk), used for between-group comparison, was −4.6 ± 4.5 in the SAGX group compared with −0.6 ± 4.0 in the saw palmetto group, indicating a statistically significant difference (*p* = 0.031, Wilcoxon rank-sum test; Table 2). Importantly, the magnitude of reduction in the SAGX group exceeded the minimal clinically important difference (MCID) of 3 points, indicating a clinically meaningful improvement.

#### 3.2.2. IPSS Subscores

Frequency

Urination frequency decreased from 2.6 ± 1.0 at baseline to 1.4 ± 1.0 at Week 12 in the SAGX group, whereas the saw palmetto group showed a negligible change from 2.0 ± 0.7 to 2.2 ± 1.5 (Figure 2B). The mean change in frequency score (Δ12 wk) was −1.2 ± 0.7 in the SAGX group compared with 0.2 ± 1.4 in the saw palmetto group, demonstrating a significantly greater improvement with SAGX (*p* = 0.006, Wilcoxon rank-sum test; Table 2).

Storage

The IPSS storage score, defined as the sum of urinary frequency, urgency, and nocturia subscores, decreased from 6.2 ± 1.7 at baseline to 3.5 ± 1.5 at Week 12 in the SAGX group, while the saw palmetto group showed minimal change from 5.6 ± 1.6 to 5.4 ± 2.7 (Figure 2C). The mean change in storage score (Δ12 wk) was −2.7 ± 1.6 in the SAGX group compared with −0.2 ± 1.9 in the saw palmetto group, indicating a significantly greater improvement in storage symptoms with SAGX (*p* = 0.003, Wilcoxon rank-sum test; Table 2).

Quality of life

Quality-of-life (QoL) scores improved from 3.1 ± 1.1 at baseline to 2.3 ± 0.9 at Week 12 in the SAGX group, whereas the saw palmetto group showed a slight worsening from 3.0 ± 1.2 to 3.3 ± 1.5 (Figure 2D). The mean change in QoL score (Δ12 wk) was −0.8 ± 1.2 in the SAGX group and 0.3 ± 1.1 in the saw palmetto group, representing a significant improvement with SAGX (*p* = 0.035, Wilcoxon rank-sum test; Table 2).

Taken together, SAGX improved the IPSS total score, the QoL and frequency subscores, as well as the composite storage score. In contrast, changes in composite voiding symptoms and other individual subscores did not differ significantly between groups. The results of primary and secondary outcomes related to IPSS scores and subscores are summarized in Table 2.

### 3.3. Secondary Endpoints

#### 3.3.1. Total IIEF Score

**Figure 3:**
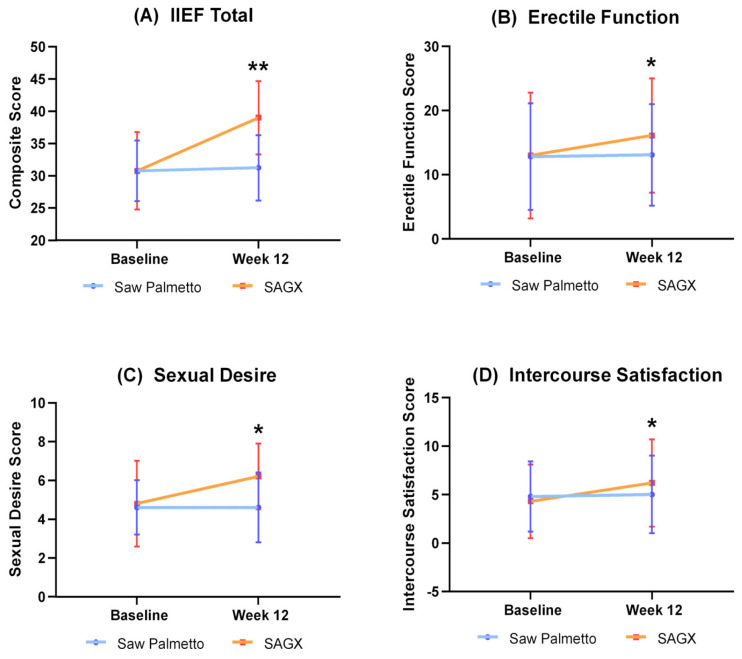
Changes in International Index of Erectile Function (IIEF) scores in each group (PPS, mean ± SEM; vertical red and blue lines represent SEM). (**A**) IIEF total score from baseline to Week 12 (*p* = 0.005). (**B**) Erectile function score from baseline to Week 12 (*p* = 0.013). (**C**) Sexual desire score from baseline to Week 12 (*p* = 0.025). (**D**) Intercourse satisfaction score from baseline to Week 12 (*p* = 0.043). * *p* < 0.05, ** *p* < 0.01, based on between-group changes from the baseline (Δ12 wk), Wilcoxon rank-sum test. Higher scores indicate improvement.

At baseline, overall IIEF scores were comparable between groups. In the SAGX group, scores increased from 30.8 ± 21.7 at baseline to 39.0 ± 20.4 at Week 12, whereas the saw palmetto group showed minimal change from 30.8 ± 16.3 to 31.9 ± 17.5 (Figure 3A).

The mean change in total IIEF score (Δ12 wk) was +8.2 ± 5.1 in the SAGX group compared with +1.1 ± 7.7 in the saw palmetto group, indicating a statistically significant improvement with SAGX (*p* = 0.005, Wilcoxon rank-sum test; Table 3).

#### 3.3.2. IIEF Subscores

Erectile Function

Erectile function scores increased from 13.0 ± 9.8 at baseline to 16.1 ± 8.9 at Week 12 in the SAGX group, whereas the saw palmetto group showed minimal change from 12.8 ± 8.3 to 13.1 ± 7.9 (Figure 3B). The mean change in erectile function score (Δ12 wk) was +3.1 ± 2.5 in the SAGX group compared with +0.3 ± 2.4 in the saw palmetto group, indicating a significantly greater improvement with SAGX (*p* = 0.013, Wilcoxon rank-sum test; Table 3).

Sexual Desire and Intercourse Satisfaction

Sexual desire scores increased from 4.8 ± 2.2 at baseline to 6.1 ± 1.7 at Week 12 in the SAGX group, whereas the saw palmetto group showed no appreciable change from 4.6 ± 1.4 to 4.6 ± 1.8 (Figure 3C). The mean change in sexual desire score (Δ12 wk) was +1.3 ± 1.3 in the SAGX group compared with 0.0 ± 1.3 in the saw palmetto group, demonstrating a significant between-group difference (*p* = 0.025, Wilcoxon rank-sum test; Table 3).

Intercourse satisfaction scores increased from 4.3 ± 3.8 at baseline to 6.1 ± 4.5 at Week 12 in the SAGX group, while the saw palmetto group showed minimal change from 4.8 ± 3.6 to 5.0 ± 4.0 (Figure 3D). The mean change in intercourse satisfaction score (Δ12 wk) was +1.8 ± 1.5 in the SAGX group compared with +0.2 ± 2.2 in the saw palmetto group, indicating a significantly greater improvement with SAGX (*p* = 0.043, Wilcoxon rank-sum test; Table 3).

Overall, patients taking SAGX demonstrated improvements in the total IIEF score as well as in the erectile function, sexual desire, and intercourse satisfaction subscores. In contrast, changes in orgasmic function and overall satisfaction did not differ significantly between groups. Detailed changes in IIEF scores are summarized in Table 3.

It should be noted that while statistically significant between-group differences were observed for IIEF total score, erectile function, sexual desire, and intercourse satisfaction, the remaining subscores of orgasmic function and overall satisfaction did not reach statistical significance. These non-significant trends should be interpreted cautiously, particularly given the small sample size and the high inter-individual variability inherent to self-reported outcome measures in this population.

### 3.4. Safety Assessment

In the SAGX group, a total of six adverse events (AEs) were reported in three subjects (counting each subject once): epigastric discomfort (one case/one subject), dizziness (two cases/one subject), aggravation of nocturia (two cases/one subject), and pruritus (one case/one subject). All events were mild in intensity, and two events—epigastric discomfort and pruritus—were considered possibly related to the investigational product.

In the saw palmetto group, a total of 11 AEs were reported in seven subjects (counting each subject once), including myalgia (two cases/two subjects), fatigue (one case/one subject), acute gout (two cases/one subject), dyspepsia (one case/one subject), testicular discomfort (one case/one subject), aggravation of nocturia (one case/one subject), and pruritus (one case/one subject). Acute gout, which occurred in a single subject, was assessed as mild to moderate in severity but not related to the investigational product according to the investigator’s judgment. All other adverse events were mild, and three events—fatigue, dyspepsia, and pruritus—were considered possibly related to the investigational product.

In the saw palmetto group, one subject exhibited mild elevations in AST and ALT (one case each), which were considered possibly related to the investigational product. In contrast, no treatment-related hematologic or blood chemistry abnormalities were observed in the SAGX group. All adverse events are summarized in Table 4.

## 4. Discussion

The prevalence of urological disorders increases with age, becoming particularly prominent in middle-aged and older men. Among these conditions, lower urinary tract symptoms (LUTS) substantially impair quality of life, largely due to oxidative stress-induced cellular damage and the resulting inflammatory processes that are known to drive symptoms’ progression [4]. SAGX has been shown to mitigate these pathological mechanisms by activating the Nrf2/HO-1 pathway, thereby reducing reactive oxidative species (ROS) levels and restoring homeostatic balance within the lower urinary tract [17]. Consistent with these mechanistic findings, previous clinical studies have demonstrated that SAGX significantly improved prostate function in men with LUTS [20].

Accordingly, this study was conducted as a pilot clinical trial aimed at establishing preliminary evidence regarding the comparative efficacy and safety of SAGX and commercially available saw palmetto extract in men with LUTS. A 12-week, randomized, double-blind, parallel-group design was used to determine whether SAGX exhibits sufficient therapeutic potential in comparison with saw palmetto to justify subsequent large-scale confirmatory clinical trials.

After 12 weeks of intake, the SAGX group demonstrated significantly greater improvements than the saw palmetto group in several key clinical outcomes, including the IPSS total score (*p* = 0.031), urinary frequency (*p* = 0.006), quality of life (*p* = 0.035), and storage symptoms (*p* = 0.003). In the context of LUTS management, the minimum clinically important difference (MCID) is generally defined as a reduction of 3 points in the IPSS [25]. The mean reduction of 4.6 points observed in the SAGX group exceeded this threshold, indicating a clinically meaningful therapeutic effect. In contrast, the saw palmetto group exhibited only a 0.6-point reduction, a magnitude that falls below the MCID and is considered clinically negligible. This minimal change aligns with findings from large-scale trials such as CAMUS and STEP, which have reported saw palmetto to be largely indistinguishable from a placebo, thereby indirectly reaffirming those results in the present smaller-scale study [6,7]. The superior efficacy observed with SAGX may be attributable to its distinct mechanistic profile. Whereas saw palmetto is thought to exert its effects primarily through the inhibition of 5α-reductase, SAGX contains high levels of bioactive tanshinones that possess potent antioxidant properties and are known to activate the Nrf2/HO-1 signaling pathway [17]. Activation of this pathway may reduce prostatic inflammation and mitigate oxidative stress within the bladder, mechanisms that plausibly contribute to the observed improvements in storage-related symptoms, most notably urinary frequency.

Saw palmetto (*Serenoa repens*) remains the most widely utilized phytotherapeutic agent for the management of benign prostatic hyperplasia (BPH) and associated LUTS. However, our findings of a negligible 0.6-point IPSS reduction in the active control group are consistent with landmark trials such as STEP and CAMUS, which demonstrated that saw palmetto often does not differ significantly from a placebo in improving total IPSS scores or subscores related to storage and voiding symptoms [6,7]. At the 12-week interim analysis of the STEP trial, for example, the mean IPSS reduction was only 0.7 points. A potential factor in these results is the clinical onset of action for androgen-dependent pathways. Standard 5-α-reductase inhibitors (5-ARIs), which share a theorized mechanism with saw palmetto, typically require 6 to 12 months of administration to achieve significant prostate volume reduction and symptomatic relief [26,27]. Consequently, the 12-week duration of the present study may have been insufficient for the saw palmetto group to reach a therapeutic threshold. This contrast further highlights the supportive potential of SAGX, whose efficacy likely stems from the rapid activation of antioxidant and microcirculatory pathways (Nrf2/HO-1). Targeting these oxidative and inflammatory drivers may provide more immediate symptomatic relief than traditional androgen-focused pathways, particularly within a short 12-week window.

A particularly noteworthy finding of this study is the significant improvement in erectile function, as reflected by increases in IIEF scores among men treated with SAGX (*p* = 0.005). LUTS and erectile dysfunction (ED) are known to share common pathophysiological pathways, including pelvic atherosclerosis and endothelial dysfunction [28,29]. The vasodilatory and microcirculatory-enhancing properties of SAGX may therefore augment pelvic perfusion, offering a plausible mechanistic basis for the concurrent improvement in urinary and sexual symptoms observed in this study [30]. This stands in contrast to 5-α-reductase inhibitors, which are well documented to exacerbate ED.

Although statistical significance was not reached for several secondary symptom domains, most numerical changes favored SAGX in both the IPSS and IIEF subscores. Given the limited sample size and exploratory design, the study may have been underpowered to detect smaller but potentially clinically relevant between-group differences. Thus, these non-significant trends should be interpreted cautiously, particularly given the small sample size and the high inter-individual variability inherent to self-reported outcome measures in this population.

Collectively, the findings from this study indicate that SAGX is well tolerated and was associated with fewer reported adverse events, which may translate into more substantial supportive benefits for prostate health than saw palmetto extract. Given its favorable preliminary efficacy and safety characteristics, SAGX may constitute a valuable option for supporting improvements in urinary symptoms and sexual function in men.

This study has several limitations. First, the small sample size (*N* = 30) characterizes this as a pilot study; thus, the results should be interpreted as hypothesis-generating rather than definitive. Second, the analysis was restricted to the per-protocol set, which may introduce attrition bias. Third, the use of patient-reported outcome measures such as IPSS and IIEF may introduce inter-individual variability in symptom scoring. Fourth, the short study period (12 weeks) precludes the assessment of long-term efficacy, safety, and disease progression. In particular, the long-term safety profile and potential drug–herb interactions in elderly patients with comorbidities and concomitant medications could not be fully evaluated in the present study, which limits the generalizability of our safety findings. *Salvia miltiorrhiza* is known to inhibit CYP1A2 and CYP2C family enzymes, potentially increasing plasma concentrations of co-administered drugs metabolized by these pathways, most notably warfarin [31,32,33]. Patients on anticoagulant or antiplatelet therapy were excluded from this study (Exclusion Criterion 12). However, a broader elderly male population may include individuals on such medications, and the safety of SAGX in this subgroup requires dedicated evaluation in future pharmacokinetic interaction studies and extended-duration confirmatory trials. Fifth, the lack of a placebo group limits the interpretation and conclusions of the study, thus necessitating a large-scale follow-up study.

Post hoc power analysis based on the observed effect size (Cohen’s d = 0.94) for the primary endpoint was utilized to inform future research. A definitive three-arm confirmatory trial—comparing SAGX, saw palmetto, and placebo—would require approximately 22 participants per arm (66 total) to maintain 80% power at α = 0.05. Accounting for an anticipated 25% dropout rate, a total enrollment of 90 participants (30 per group) is recommended to substantiate these preliminary results. Furthermore, subsequent well-organized projects should feature comparative phytochemical profiling and characterization of specific active metabolic substrates for both extracts to fully elucidate their distinct pharmacological mechanisms. Future studies with larger cohorts, intent-to-treat (ITT) analysis, placebo controls, long-term follow-up, and comprehensive safety assessments are warranted to validate these promising findings.

## 5. Conclusions

In this 12-week pilot study, SAGX demonstrated statistically significant and clinically meaningful improvements in IPSS total score, storage symptoms, quality of life, and erectile function compared with saw palmetto. While these hypothesis-generating findings are limited by the small sample size and exploratory design, they provide a robust quantitative foundation for future research. Based on the observed large effect size (Cohen’s d = 0.94), a definitive three-arm confirmatory trial (SAGX vs. saw palmetto vs. placebo; *N* = 90, accounting for 25% attrition) is warranted to validate these preliminary signals.

## Figures and Tables

**Table 1:** Demographics of participants completing the study.

Characteristic	SAGX (*N* = 13)	Saw Palmetto (*N* = 12)	*p*-Value ^W^
Age (years)	67.0 ± 6.8	67.4 ± 6.9	0.473
Height (cm)	169.1 ± 8.8	167.0 ± 4.3	0.246
Weight (kg)	65.7 ± 8.3	69.8 ± 8.7	0.234
Blood pressure			
SBP	121.5 ± 11.4	126.5 ± 9.0	0.176
DBP	75.4 ± 9.8	80.8 ± 9.7	0.660

^W^ Between-group analysis (Wilcoxon rank sum test).

**Table 2:** Changes in IPSS total and subscores from baseline to Week 12.

		SAGX (*N* = 13)	Saw Palmetto (*N* = 12)	*p*-Value ^W^
IPSS total	0 wk	14.1 ± 4.1	12.7 ± 4.4	0.366
	Δ12 wk	−4.6 ± 4.5	−0.6 ± 4.0	0.031
Incomplete emptying	0 wk	1.8 ± 1.1	1.5 ± 1.1	0.447
	Δ12 wk	−0.2 ± 0.9	−0.3 ± 0.9	0.958
Urinary frequency	0 wk	2.6 ± 1.0	2.0 ± 0.7	0.105
	Δ12 wk	−1.2 ± 0.7	0.2 ± 1.4	0.006
Intermittency	0 wk	1.8 ± 1.0	1.4 ± 1.1	0.310
	Δ12 wk	−0.5 ± 0.8	0.0 ± 0.9	0.170
Urgency	0 wk	2.0 ± 1.1	1.9 ± 0.8	0.829
	Δ12 wk	−0.8 ± 0.6	−0.4 ± 0.9	0.257
Weak stream	0 wk	2.4 ± 1.4	2.5 ± 1.0	0.815
	Δ12 wk	−0.6 ± 1.1	−0.2 ± 1.4	0.384
Straining	0 wk	1.8 ± 1.6	1.7 ± 1.2	0.859
	Δ12 wk	−0.6 ± 1.4	0.0 ± 0.7	0.199
Nocturia	0 wk	1.6 ± 0.7	1.7 ± 0.8	0.859
	Δ12 wk	−0.8 ± 0.9	0.1 ± 0.5	0.101
Quality of life	0 wk	3.1 ± 1.1	3.0 ± 1.2	0.778
	Δ12 wk	−0.8 ± 1.2	0.3 ± 1.1	0.035
Voiding ^1^	0 wk	7.8 ± 3.2	7.0 ± 3.3	0.526
	Δ12 wk	−2.7 ± 1.6	−0.4 ± 1.9	0.103
Storage ^2^	0 wk	6.2 ± 1.7	5.6 ± 1.6	0.503
	Δ12 wk	−2.7 ± 1.6	−0.2 ± 1.9	0.003

Δ Score changes from the baseline; data are presented as the mean ± standard deviation. ^W^ Between-group analysis (Wilcoxon rank-sum test). ^1^ Sum of incomplete emptying, intermittency, weak stream and straining subscores. ^2^ Sum of frequency, urgency, and nocturia subscores.

**Table 3:** Changes in IIEF total and subscores from baseline to Week 12.

		SAGX (*N* = 13)	Saw Palmetto (*N* = 12)	*p*-Value ^W^
IIEF total	0 wk	30.8 ± 21.7	30.8 ± 16.3	0.978
	Δ12 wk	8.2 ± 5.1	1.1 ± 7.7	0.005
Erectile function	0 wk	13.0 ± 9.8	12.8 ± 8.3	0.964
	Δ12 wk	3.1 ± 2.5	0.3 ± 2.4	0.013
Orgasmic function	0 wk	3.9 ± 4.0	3.6 ± 2.8	0.808
	Δ12 wk	1.2 ± 1.3	0.3 ± 1.4	0.108
Sexual desire	0 wk	4.8 ± 2.2	4.6 ± 1.4	0.732
	Δ12 wk	1.3 ± 1.3	0.0 ± 1.3	0.025
Intercourse satisfaction	0 wk	4.3 ± 3.8	4.8 ± 3.6	0.728
	Δ12 wk	1.8 ± 1.5	0.2 ± 2.2	0.043
Overall satisfaction	0 wk	4.7 ± 2.4	4.9 ± 2.0	0.803
	Δ12 wk	0.7 ± 1.2	−0.2 ± 1.4	0.152

Δ Score changes from the baseline. Data are presented as the mean ± standard deviation. ^W^ Between-group comparison (Wilcoxon rank sum test).

**Table 4:** Adverse Events.

Category of Adverse Event	SAGX Group	Saw Palmetto Group
Musculoskeletal System		
Myalgia		2 cases/2 subjects
Fatigue		1 case/1 subject ^(1)^
Gastrointestinal System		
Dyspepsia		1 case/1 subject ^(1)^
Heartburn	1 case/1 subject	
Nervous System		
Dizziness	2 cases/1 subject	
Genitourinary System		
Testicular discomfort		1 case/1 subject
Aggravation of nocturia	2 cases/1 subject ^(3)^	1 case/1 subject
Dermatologic System		
Pruritus	1 case/1 subject ^(3)^	1 case/1 subject ^(1)^
Blood chemistry tests		
ALT elevation		1 case/1 subject ^(2)^
AST elevation		1 case/1 subject ^(2)^
Total	6 cases/3 subjects	11 cases/7 subjects

^(1)~(3)^ Duplicate subjects.

## Data Availability

The data will be made available based on request to the corresponding author and will not be publicly available due to privacy and ethical reasons.

## Supplementary Materials

The following supporting information can be downloaded at https://www.mdpi.com/article/10.3390/nu18111752/s1. Supplement S1. IPSS Questionnaire; Supplement S2. IIEF Questionnaire.

## Abbreviations

The following abbreviations are used in this manuscript.

AE	Adverse event
ALT	Alanine transaminase
AST	Aspartate aminotransferase
AUA	American Urological Association
BPH	Benign prostatic hyperplasia
DBP	Diastolic blood pressure
HO-1	Heme oxygenase 1
IIEF	International Index of Erectile Function
IPSS	International Prostate Symptom Score
LOCF	Last observation carried forward
LUTS	Lower urinary tract symptom
MFDS	Ministry of Food and Drug Safety
Nrf2	Nuclear factor erythroid 2-related factor 2
PPS	Per protocol set
PSA	Prostate-specific antigen
QoL	Quality of life
ROS	Reactive oxidative species
SAE	Serious adverse event
SAGX	*Salvia miltiorrhiza* extract
SBP	Systolic blood pressure
