# Supplementary material for: Efficacy and Safety of Salvia miltiorrhiza Extract (SAGX) Compared with Saw Palmetto in Men with Lower Urinary Tract Symptoms: A 12-Week, Randomized, Double-Blind, Parallel-Group Pilot Study
Source: Nutrients. 2026 May 29;18(11):1752. doi: 10.3390/nu18111752 (PMC13258752; doi:10.3390/nu18111752)
Supplement: Supplementary file 1 [file nutrients-18-01752-s001.zip › nutrients-4293086-supplementary.pdf]

Supplement S1. IPSS Questionnaire

International Prostate Symptom Score (IPSS) Questionnaire

Screening Number	S ___ ___ ___ (Subject Code Number)	Subject Initials	___ ___ ___
Assessment Date	Y: 20__ M : _ D : __	Visit Number	☐ V1 ☐ V2<br>☐ Additional Visit

◈ Please consider your urinary symptoms during the past 1 month. For each question below, select the response that best represents how often the symptom occurred when you urinated.

International Prostate Symptom Score (IPSS)						
	Not at all	Less than 1 time in 5	1-2 times in 5	2-3 times in 5	3-4 times in 5	Almost always
1. During the past month, how often have you had the sensation that your bladder was not completely empty after you finished urinating?	0	1	2	3	4	5
2. During the past month, how often have you had to urinate again within 2 hours after finishing urination?	0	1	2	3	4	5
3. During the past month, how often have you found that you stopped and started again several times when urinating?	0	1	2	3	4	5
4. During the past month, how often have you found it difficult to postpone urination?	0	1	2	3	4	5
5. During the past month, how often have you had a weak urinary stream?	0	1	2	3	4	5
6. During the past month, how often have you had to strain or push to begin urination?	0	1	2	3	4	5
7. During the past month, how many times did you typically get up during the night to urinate from the time you went to bed until the time you got up in the morning?	None	Once	Twice	3 times	4 times	5 times or more
	0	1	2	3	4	5
**Total Symptom Score ____pts**						

Quality of Life							
	Delighted	Pleased	Mostly satisfied	Mixed (equally satisfied and dissatisfied	Mostly dissatisfied	Miserable	Intolerable
If you were to spend the rest of your life with your urinary condition exactly as it is now, how would you feel about that?	0	1	2	3	4	5	6
**Quality of Life Score ______pts**							

**Subject Signature**	

Supplement S2. IIEF Questionnaire

International Index of Erectile Function (IIEF) Questionnaire

**Screening Number**	0 1 - S ___ ___ ___ (Site Code / Serial Number)	**Subject Initials**	___ ___ ___
**Assessment Date**	Y: 20__ M:___ D: ___	**Visit Number**	☐ V2 ☐ V3 ☐ V4 ☐ Additional Visit

◈ **Please read the following questions and mark the number that best applies.**

1. During the past 4 weeks, how often were you able to get an erection during sexual activity?

⓪ No sexual activity

① Almost never or never.

② A few times (much less than half the time)

③ Sometimes (about half the time)

④ Most times (much more than half the time)

⑤ Almost always or always

2. During the past 4 weeks, when you had erections with sexual stimulation, how often were your erections hard enough for penetration?

⓪ No sexual activity

① Almost never or never.

② A few times (much less than half the time)

③ Sometimes (about half the time)

④ Most times (much more than half the time)

⑤ Almost always or always

3. During the past 4 weeks, when you attempted sexual intercourse, how often were you able to penetrate your partner?

⓪ No sexual activity

① Almost never or never.

② A few times (much less than half the time)

③ Sometimes (about half the time)

④ Most times (much more than half the time)

⑤ Almost always or always

4. During the past 4 weeks, during sexual intercourse, how often were you able to maintain your erection after penetration?

⓪ No sexual activity

① Almost never or never.

② A few times (much less than half the time)

③ Sometimes (about half the time)

④ Most times (much more than half the time)

⑤ Almost always or always

5. During the past 4 weeks, during sexual intercourse, how difficult was it to maintain your erection until completion of intercourse?

⓪ No sexual activity

① Almost never or never.

② A few times (much less than half the time)

③ Sometimes (about half the time)

④ Most times (much more than half the time)

⑤ Almost always or always

6. During the past 4 weeks, how many times did you attempt sexual intercourse?

⓪ No sexual activity

① Almost never or never.

② A few times (much less than half the time)

③ Sometimes (about half the time)

④ Most times (much more than half the time)

⑤ Almost always or always

7. During the past 4 weeks, when you attempted sexual intercourse, how often was it satisfactory for you?

⓪ No sexual activity

① Almost never or never.

② A few times (much less than half the time)

③ Sometimes (about half the time)

④ Most times (much more than half the time)

⑤ Almost always or always

8. During the past 4 weeks, how enjoyable was sexual intercourse for you?

⓪ No sexual activity

① Almost never or never.

② A few times (much less than half the time)

③ Sometimes (about half the time)

④ Most times (much more than half the time)

⑤ **Almost always or always**

9. During the past 4 weeks, when you had sexual stimulation or intercourse, how often did you ejaculate?

⓪ **No sexual activity**

① **Almost never or never.**

② **A few times (much less than half the time)**

③ **Sometimes (about half the time)**

④ **Most times (much more than half the time)**

⑤ **Almost always or always**

10. During the past 4 weeks, when you had sexual stimulation or intercourse, how often did you experience orgasm (climax), whether or not you ejaculated?

⓪ **No sexual activity**

① **Almost never or never.**

② **A few times (much less than half the time)**

③ **Sometimes (about half the time)**

④ **Most times (much more than half the time)**

⑤ **Almost always or always**

11. During the past 4 weeks, how often did you feel sexual desire?

⓪ **No sexual activity**

① **Almost never or never.**

② **A few times (much less than half the time)**

③ **Sometimes (about half the time)**

④ **Most times (much more than half the time)**

12. During the past 4 weeks, how would you rate your level of sexual desire?

⓪ **No sexual activity**

① **Almost never or never.**

② **A few times (much less than half the time)**

③ **Sometimes (about half the time)**

④ **Most times (much more than half the time)**

13. During the past 4 weeks, how satisfied have you been with your overall sex life?

⓪ **No sexual activity**

① **Almost never or never.**

② **A few times (much less than half the time)**

③ **Sometimes (about half the time)**

④ **Most times (much more than half the time)**

14. During the past 4 weeks, how satisfied have you been with your sexual relationship with your partner?

⓪ **No sexual activity**

① **Almost never or never.**

② **A few times (much less than half the time)**

③ **Sometimes (about half the time)**

④ Most times (much more than half the time)

15. During the past 4 weeks, how would you rate your confidence that you could get and maintain an erection?

◎ No sexual activity

① Almost never or never.

② A few times (much less than half the time)

③ Sometimes (about half the time)

④ Most times (much more than half the time)

Subject Signature	
